# Decellularization of Wharton’s Jelly Increases Its Bioactivity and Antibacterial Properties

**DOI:** 10.3389/fbioe.2022.828424

**Published:** 2022-03-11

**Authors:** M. Dubus, L. Scomazzon, J. Chevrier, A. Montanede, A. Baldit, C. Terryn, F. Quilès, C. Thomachot-Schneider, S. C. Gangloff, N. Bouland, F. Gindraux, H. Rammal, C. Mauprivez, H. Kerdjoudj

**Affiliations:** ^1^ Biomatériaux et Inflammation en Site Osseux (BIOS) EA 4691, Université de Reims Champagne Ardenne, Reims, France; ^2^ UFR d’Odontologie, Université de Reims Champagne Ardenne, Reims, France; ^3^ Laboratoire d’étude des Microstructures et de Mécanique des Matériaux (LEM3), UMR CNRS 7239, Université de Lorraine, Metz, France; ^4^ Plateau Technique PICT, Université de Reims Champagne Ardenne, Reims, France; ^5^ CNRS, LCPME, Université de Lorraine, Nancy, France; ^6^ Groupe d’Étude des Géomatériaux et Environnement Naturels, Anthropiques et Archéologiques (GEGENAA), Université de Reims Champagne Ardenne, Reims, France; ^7^ UFR de Pharmacie, Université de Reims Champagne Ardenne, Reims, France; ^8^ Service d’anatomopathologie, UFR de Médecine, Université de Reims Champagne Ardenne, Reims, France; ^9^ Laboratoire de Nanomédecine, Imagerie, Thérapeutique, Université Bourgogne Franche-Comté, Besançon, France; ^10^ Centre Hospitalier Universitaire de Reims, Pôle Médecine Bucco-dentaire, Hôpital Maison Blanche, Reims, France

**Keywords:** Wharton’s jelly, decellularization, biocompatibility, bioactivity, antibacterial, *in vivo*

## Abstract

The field of regenerative medicine has recently seen an emerging trend toward decellularized extracellular matrix (ECM) as a biological scaffold for stem cell-delivery. Human umbilical cord represents a valuable opportunity from both technical and ethical point of view to obtain allogenic ECM. Herein, we established a protocol, allowing the full removal of cell membranes and nuclei moieties from Wharton’s jelly (WJ) tissue. No alterations in the ECM components (*i.e.,* collagen, GAG content, and growth factors), physical (*i.e.,* porosity and swelling) and mechanical (*i.e.,* linear tensile modulus) properties were noticed following WJ processing. Furthermore, no effect of the tissue processing on macromolecules and growth factors retention was observed, assuring thus a suitable bioactive matrix for cell maintenance upon recellularization. Based on the *in vitro* and *in vivo* biodegradability and stromal cell homing capabilities, decellularized WJ could provide an ideal substrate for stromal cells adhesion and colonization. Interestingly, the tissue processing increased the antibacterial and antiadhesive properties of WJ against *Staphylococcus aureus* and *Staphylococcus epidermidis* pathogens. Altogether, our results indicate that decellularized WJ matrix is able to limit Staphylococcus-related infections and to promote stromal cell homing, thus offering a versatile scaffold for tissue regenerative medicine.

## 1 Introduction

With a length and a diameter of about 40 and 1.5 cm, the umbilical cord connects the placenta to the developing fetus, ensuring thereby its nourishment, and oxygenation. The umbilical cord stroma contains a unique stem cell-rich gelatinous substance called Wharton’s jelly (WJ). Ensuring the umbilical cord flexibility, the major role of WJ is the resistance to torsional, and compressive stresses imposed upon the umbilical vessels during fetal development (Pennati, 2001). Besides its protective role, the WJ is a rich reservoir of mesenchymal stromal cells and growth factors, and contains significant amounts of extracellular matrix (ECM) components such as collagen, hyaluronic acid, and several glycosaminoglycans (Nanaev et al., 1997; Franc et al., 1998; Sobolewski et al., 2005). With its immunologically-privileged status, the WJ lends credence to its use as an allograft for difficult-to-heal wounds. To this end, fresh or cryopreserved WJ patches have been used in clinical practice with proven reparative effects following skin graft procedure and treatment of foot ulcers and diabetic ulcers with osteomyelitis. (Caputo et al., 2016; Raphael, 2016; Marston et al., 2019; Tettelbach et al., 2019). Such an attractive combination of biomechanical, immunological and biochemical features makes WJ a versatile candidate material for the regenerative medicine field.

Regenerative medicine provides tools such as multifunctional scaffolds in the aim of avoiding bacterial infection repairing injuries. (Renth and Detamore, 2012). The effectiveness of scaffold -based therapy comes from the ability of the scaffold to recapitulate healthy tissue cues that drive tissue repair and regeneration. Several studies aim at developing innovative bio-inspired scaffolds in order to counteract ECM loss following degeneration and to support functional recovery by endogenous damaged microenvironment. In such scenario, the potential therapeutic scaffold would be the native ECM; an acellular component of tissue that provides the structural support and biochemical cues for determining a cell’s fate. (Hinderer et al., 2016; Yu et al., 2016; Gilpin and Yang, 2017; Damodaran and Vermette, 2018; Spang and Christman, 2018). Furthermore, the bloodless scaffold could become the object of bacterial adhesion, and a large number of bacterial adhesions will form a biofilm resistant matrix, making it difficult to cure, and causing a relapse in infection. To process the ECM as a tool in regenerative therapy, the excised tissue must first undergo physical, chemical and/or enzymatic treatments to remove cellular components, and nuclear moieties. Therefore, the resulting decellularized ECM is thought to be an ideal and safe system to deliver chemokines and growth factors, and to provide adequate structural, and biomechanical microarchitecture in the damaged microenvironment. (Crapo et al., 2011; Keane et al., 2015). However, the consequences of ineffective or incomplete decellularization could result in host’s foreign body reaction, which would lead to the formation of fibrous capsule. (Keane et al., 2012; Hussein et al., 2016).

Successful commercialization of any tissue product relies on the effective preservation of key biological components essential to maintain the intended therapeutic action of the tissue. The main disadvantage of WJ as a tissue source is its transientness as it is only available in the active form during a short time period immediately postpartum. An effective solution to this problem may be provided by its careful decellularization. Few studies showed that decellularized WJ provides a biocompatible framework with suitable features for cartilage, (Penolazzi et al., 2020), liver, (Kehtari et al., 2019), neural tissue (Kočí et al., 2017), and wound (Beiki et al., 2017) repair and regeneration. Despite significant reduction in DNA content following the decellularization, the reported values, of about several micrograms per milligram of dry tissue, are not in full agreement with Crapo *et al.*, suggesting values of about 50 ng/mg of dry tissue as safety threshold to avoid host’s adverse reaction. (Crapo et al., 2011). To achieve such requirement, substantial disruption of the WJ matrix (*i.e.,* powdered and hydrogel based matrix) were reported. (Jadalannagari et al., 2017; Basiri et al., 2019; Lin et al., 2020). In other studies, WJ derived products were reported in combination with diverse polymers and ceramics, enhancing the biological activities of the scaffold. (Martínez et al., 2017; Basiri et al., 2019). In the present study, a novel protocol was developed to remove all cellular components from WJ and to generate implantable and less host reactive WJ-based patches. For better storage, the resulting patches were freeze-dried and the effects of the tissue processing on the structural, biochemical and biomechanical properties were further investigated. The biological performance of WJ-based patches was assessed *in vitro* and *in vivo*. As mentioned above, WJ was successfully used to treat foot ulcers and diabetic ulcers with osteomyelitis, and its regenerative capabilities were mainly attributed to the presence of stromal cells, growth factors and hyaluronic acid. (Marston et al., 2019). The intrinsic antimicrobial properties of WJ is still an unexplored field. Herein and to our knowledge, we report for the first time, the antibacterial properties of decellularized WJ-based patches, opening the route for the development of new and affordable WJ-bioactive antibacterial matrix for regenerative medicine.

## 2 Material and Methods

### 2.1 Wharton’s Jelly Preparation

#### 2.1.1 Wharton’s Jelly Collection

Human umbilical cord harvesting was approved ethically and methodologically by our local Research Institution and was conducted with informed patients (written consent) in accordance with the usual ethical legal regulations (Article R 1243-57). All procedures were done in accordance with our authorization and registration number DC-2014-2262 given by the National “Cellule de Bioéthique”. Fresh human umbilical cords, obtained after full-term births, were washed several times with Phosphate Buffered Saline (PBS, Gibco, and France) to remove blood components, dissected, and vascular structures removed. Wharton’s jelly matrix (WJ) was then peeled off the amniotic surrounding membrane, and preserved at -20°C.

#### 2.1.2 Wharton’s Jelly Decellularization

After two cycles of freezing/defrosting (−20°C/20°C), samples were subjected to a decellularization protocol comprising hypotonic treatment with 1% Triton X-100 in distilled water (VWR, France) for 1 h then enzymatic treatment with 0.2 mg/ml of DNase (Sigma, France) at 37°C for 24 h under stirring. All processing residuals were removed by rinsing twice with PBS for 10 min under stirring. Finally, decellularized Wharton’s jelly (d-WJ) samples were frozen at −20°C then −80°C before freeze-drying process. Freeze-dried devitalized WJ (WJ) was used as control.

### 2.2 Effectiveness of Decellularization Processing

#### 2.2.1 DAPI Staining

The efficiency of the decellularization protocol was verified by nuclei staining with 4,6-diamidino-2-phenylindole (DAPI, 100 ng/ml, 1:3,000 dilution) for 5 min and fluorescence microscopy imaging (Axiovert 200 M microscope, Zeiss, Oberkochen, Germany, Objective ×10).

#### 2.2.2 DNA Quantification

DNA was extracted from samples using MasterPure^TM^ DNA Purification Kit (Epicentre® Biotechnologies) in accordance with the manufacturer protocol. Freeze-dried samples were weighed prior to DNA extraction. Extracted DNA was then assessed by measuring the absorbance at 260 and 280 nm (Nanodrop®, Thermo Scientific) with 260/280 nm absorbance ratio for all measured samples comprised between 1.8 and 2. DNA concentration was calculated according to tissue weight (ng of DNA/μL/mg of dry tissue).

#### 2.2.3 Histology

Samples were embedded in paraffin and cut into 4 μm sections (rotation microtome RM 2055, Leica Microsystems). Hematoxylin-eosin-safran (HES), Blue Alcian (at pH 1.5 and 2.5) and Masson’s trichrome (MT) stainings were performed separately on consecutive tissue sections and images were taken using VS 120 OLYMPUS scanner.

#### 2.2.4 Collagen Quantification

Total collagen content of samples was evaluated using a colorimetric total collagen assay kit (BioVision, France) in accordance with the manufacturer protocol. Briefly, samples were homogenized with distilled water and hydrolyzed with 12 M HCl for 3 h at 120°C. Hydrolyzed samples (15 μl) were transferred in a 96-well plate and were evaporated then 100 µl of the Chloramine T reagent were added to each sample. After 5 min, 100 µl of the p dimethylaminobenzaldehyde reagent were added to each well and the plate was incubated for 90 min at 60°C. Finally, the absorbance was measured at 560 nm using a microplate reader (FLUOstar Omega microplate reader, BMG Labtech). The total collagen concentration was calculated using kit standard curve and normalized according to the weight of dry sample (mg of collagen/mg of dry tissue).

#### 2.2.5 Glycosaminoglycans Quantification

Sulphated and non-sulphated glycosaminoglycans (GAGs) were quantified in samples using a colorimetric Alcian Blue method. Briefly, 1 ml of 0.5% (w/v) Alcian Blue 8GX (Sigma, France) in HCl 0.1 M (pH 1.5 or pH 2.5) was added to samples and incubated overnight on orbital shaker (300 rpm). After centrifugation (10 min at 12,000 *g*), supernatant was removed and 250 µl of HCl 6 M were added to samples, incubated overnight on orbital shaker at room temperature and the absorbance of the extracted Alcian Blue was measured at 600 nm. The concentrations of sulphated and non-sulphated GAGs were determined, respectively, according to a chondroitin sulfate B sodium (Sigma) standard range (Alcian blue pH 1.5), and a hyaluronic acid (RenovHyal®) standard range (Alcian blue 2.5). GAG concentrations were normalized according to the weight of dry sample (µg of GAG/mg of dry tissue).

#### 2.2.6 Infrared Spectroscopy

Spectra were recorded in reflection mode between 4,000 and 800 cm^−1^ on a Bruker Vertex 70v spectrometer equipped with a Hyperion 2000 microscope and a ×15 objective controlled by the OPUS 7.5 software. A KBr beam splitter and a MCT detector were used. The resolution of the single beam spectra was 4 cm^−1^. The number of bidirectional double-sided interferogram scans was 64, which corresponds to a 40 s accumulation. All interferograms were Fourier processed using the Mertz phase correction mode and a Blackman-Harris three-term apodization function. Measurements were performed at 21 ± 1°C in an air-conditioned room. Water vapor subtraction was performed. Masks of 300 × 220 µm were used to record 58 spectra located at different areas of the freeze-dried samples. An average spectrum was then calculated from the 58 spectra of every sample, and baseline was corrected on the average spectra by concave elastic correction (4 iterations, 64 baseline points) between 4,000 and 800 cm^−1^ before further analysis of the average spectra.

### 2.3 Physical and Mechanical Features

#### 2.3.1 Scanning Electron Microscopy

Freeze-dried samples were sputtered with a thin gold–palladium film using a JEOL ion sputter JFC 1100 instrument. Scanning electron microscopy with a field emission gun (FEG-SEM) investigations were performed with a FEG-SEM (JEOL JSM-7900F, France), and images were acquired from secondary electrons at primary beam energy at 5 kV.

#### 2.3.2 Second Harmonic Generation Imaging

Two-photon excitation laser scanning confocal microscopy and second harmonic generation (SHG) were performed under circular polarization. Images of PBS- rehydrated samples were obtained with a confocal microscope (LSM 710-NLO, Carl Zeiss SAS, and Germany) coupled with CHAMELEON femtosecond Titanium-Sapphire Laser (Coherent, United States). Samples were excited at 860 nm and SHG signal was collected in 420–440 nm spectral window with ×20 objective (ON: 0.8). SHG gray level was quantified from resulting images with ImageJ.

#### 2.3.3 Mercury Intrusion Porosimetry

Pore access radii distribution and the total porosity of samples were assessed by mercury intrusion porosimetry (Micromeretics AutoPore IV 9500, Hexton, United Kingdom). The measured pore access radius ranges from 183 µm (0.003 MPa) to 0.003 µm (227 MPa). Thus, pores larger or thinner than these sizes are not considered by this technique. The incremental curve gives the mean pore radius for which the intrusive volume is maximal. The cumulative curve allows plotting the pore threshold that corresponds to the pore access allowing filling of the main part of the porous network. When both radii are close, the pore distribution can be considered as unimodal.

#### 2.3.4 Swelling Properties

Swelling ratio of samples was determined by a fluid absorption method. Freeze-dried samples were weighted and immersed in PBS for 5 min at room temperature. Hydrated samples were weighted after removing PBS excess and the equilibrium swelling ratio (Q) was calculated using the following equation,

$$Q=\frac{Wwet-Wdry}{Wdry}$$

“*W*” corresponds to the weight of samples.

#### 2.3.5 Mechanical Properties

The mechanical properties of samples were tested through quasi static tensile tests up to failure. The loading sequence was divided into two steps: 1) a dry test under elastic limits with an imposed strain around 1.6% to avoid any damages followed by 2) a PBS hydrated at 37°C one allowing a full mechanical response characterization. (Dubus et al., 2020). In between both steps, 5 min were given for the sample to accommodate prior to be tested with 1.6% strain loads and eventually up to failure. All loadings were performed at a 0.01 mm s^−1^ velocity to remain in the quasi static framework. A Universal Testing Machine Zwicky 0.5 equipped with a 10 N loadcell was used to measure samples’ response. The engineering stress and strain definition was used to process the force displacement curves and measure the linear elastic moduli.

### 2.4 Degradation Studies

*In vitro* degradation assessment of samples were performed by collagenase and hyaluronidase treatments. Freeze-dried samples (10 mg) were immersed in collagenase type II (Gibco, France) or hyaluronidase (Sigma, France) solution (1 mg/ml) for 6, 24, 48, and 72 h at 37°C. After incubation, the supernatant was discarded, and the remaining samples was freeze-dried. The mass of samples was determined before and after degradation to evaluate the percentage of remaining mass after degradation. The remaining hyaluronic acid was determined by Alcian Blue at pH 2.5 as previously described.

### 2.5 Bioactivity

#### 2.5.1 Mass Spectrometry

Freeze-dried samples were soaked in 1 ml of fetal bovine serum (FBS) free α-MEM (Lonza, France) for 72 h and the released macromolecules were analyses by mass spectrometry. Each supernatant was precipitated with DOC/TCA and resuspended in 50 µl Urea, 6M, Tris, 50 mM, and pH 8.0. From these, 20 µl were processed as follows: Cysteine residues were reduced by addition of 4 mM DTT for 45 min, alkylated by addition of IAA at 40 mM for another 45 min and IAA was blocked by addition of another 40 mM DTT for 10 min 180 µl Tris, 50 mM, CaCl_2_, and 1 mM were added together with 100 ng sequencing grade trypsin and digestion was allowed overnight at 37°C. Samples were acidified by addition of 10 µl TFA 10%. Samples were fractionated by nanoHPLC on an Ultimate 3000 system equipped with a 20 µl sample loop, a pepMap 100 C18 desalting precolumn and a 15 cm pepMap RSLC C18 fractionation column (all from Dionex). Samples (6.4 µl) were injected using the µl pickup mode and eluted by a 2–45% ACN gradient over 60 min at 300 nL/min. Fractions (340, 9 s each) were collected on a ProteineerFcII (Bruker) over 51 min and eluted fractions were directly mixed on MTP-1536 BC target (Bruker) spots to α-cyano-4-hydroxycinnamic acid (Bruker). LC-MALDI runs were processed using dedicated automatic methods piloted by WARP-LC software on an Autoflex speed MALDI-TOF/TOF mass spectrometer (Bruker), first in MS mode only, in the 700–3,500 mass range, using next-neighbour external calibration for all MALDI spots. MS runs were used for label-free relative quantification strictly as described. (Riffault et al., 2015). On this basis, masses found significantly changed between experimental groups were selected for MS/MS analysis in LIFT mode. Thereafter, all masses with S/N > 6 from one single run were also processed for LIFT fragmentation and all resulting fragmentation data were used for database interrogation. Peptide assignments were performed from TOF/TOF spectra by Mascot interrogation (Matrix Science) of the Swissprot human proteome database piloted in Mascot (Matrix Science) and compiled by Proteinscape (Bruker) with a mass tolerance of 50 ppm in TOF mode and 0.8 Da in TOF/TOF mode, with optional cysteine carbamidomethylation, methionine oxidation and with trypsin cut allowing one mis-cleavage. The minimal mascot score for peptides was set to 20 and that for proteins was set to 80. Protein change calculations and statistics were also performed strictly as previously. (Riffault et al., 2015). Identification results were cross-validated by interrogating an irrelevant database using the same criteria.

#### 2.5.2 ELISA

Freeze-dried samples were soaked in 1 ml of FBS-free α-MEM and 1 ml of α- MEM supplemented with 10% of FBS for 72 h and the released vascular endothelial growth factor (VEGF), hepatic growth factor (HGF) and transforming growth factor beta (TGF-β) were determined using human Duoset® VEGF, HGF, and TGF-β (R&D systems) respectively, according to the manufacturer’s instructions. α- MEM supplemented with 10% of FBS and incubated for 72 h at 37°C was used as control. Absorbances were measured at 450 nm using a microplate reader (FLUOstar Omega microplate reader, BMG Labtech). Released amount of growth factors was calculated from the corresponding standard curve.

### 2.6 Antibacterial Activity

For all bacterial experiments, *Staphylococcus aureus* (*S. aureus*, SH1000) and *Staphylococcus epidermidis* (*S. epidermidis,* CIP 53.124) were grown on Trypto-casein Soy (TCS) agars (Biokar) at 37°C. Both strains were then grown for 18 h in nutritive broth at 37°C and optical densities of each culture were adjusted to absorbance at 600 nm = 1 before experiments.

#### 2.6.1 Agar Diffusion Testing

Bacterial cultures were diluted at 1/500 in nutritive broth and 2 ml were poured onto a TCS agar and excess liquid was withdrawn. Freeze-dried samples (5 mm-diameter) were UV-decontaminated for 20 min, that placed onto the plate once the agar was completely dry. Plates were then incubated at 37°C for 24 h and pictures were taken to determine the zones of microbial growth inhibition.

#### 2.6.2 Bacteria Adhesion

Bacterial cultures were diluted at 1/500 in nutritive broth and 1 ml were deposited on UV-decontaminated freeze-dried samples placed into a 24-well plate. After 24 h of culture at 37°C, samples were rinsed with nutritive broth, immersed in 2 ml of nutritive broth and then sonicated for 5 min (40 kHz). Serial dilutions were further plated on TCS agars plates and the rate of viable adhered bacteria was determined after colony count.

#### 2.6.3 Confocal Laser Scanning Microscopy

For the visualization of alive and adhered *S*. *aureus* and *S. epidermidis* on samples, bacteria were labeled with Syto 9 fluorescent dye (Thermo Fischer) for 30 min. Bacteria were then imaged by confocal laser scanning microscopy (CLSM, Zeiss LSM 710 NLO, ×20 objective, Numerical Aperture 0.8, and Germany).

### 2.7 Biocompatibility

#### 2.7.1 Cell Culture

In this study, human Wharton’s jelly stromal cells (WJ-SCs) and human fibroblasts were used for *in vitro* studies. WJ-SCs were enzymatically isolated from fresh human umbilical cords obtained after full-term births and cultured in α-MEM supplemented with 10% decomplemented FBS, 1% Penicillin/Streptomycin/Amphotericin B and 1% Glutamax® (*v/v*, Gibco, France) as previously described (Mechiche Alami et al., 2014). Human gingival derived fibroblasts were isolated from gingival fragments, obtained during teeth removal Fibroblasts were cultured in DMEM-Glutamax® (Gibco, France) supplemented with 10% FBS and 1% Penicillin/Streptomycin.

#### 2.7.2 Cytotoxicity Tests

WJ-SCs and fibroblasts were cultured in 24 well chamber culture for 24 h, then 1 mg of UV-decontaminated freeze-dried samples were added in culture wells. WJ-SCs and fibroblasts cultured on tissue culture plastic without any sample were used as controls. According to the ISO/EN 10993 part 5 guideline, the cytotoxicity of sample released agents was monitored on cells by WST-1® (water-soluble tetrazolium salt-1®, Roche Diagnostics) assay and in culture supernatants by LDH (lactate dehydrogenase, Roche Diagnostics). Briefly, after 24 h of contact with samples, WST-1® was performed on cells in accordance with the manufacturer protocol, and supernatants were transferred to new culture wells before absorbance measurement at 440 nm using a FLUOstar Omega microplate reader (BMG Labtech) against a background control as blank. A wavelength of 750 nm was used as the correction wavelength. LDH activity was evaluated following the manufacturer’s instructions in cell supernatants. Absorbance was measured at 492 and 700 nm.

#### 2.7.3 Cell Proliferation Assay

Cell proliferation assay was performed with 10^4^ WJ-SCs seeded on UV-decontaminated (20 min) 5 mm-diameter-samples. WST-1® and DNA extraction were performed after 7, 10, and 15 days of culture. DNA was extracted using MasterPure^TM^ DNA Purification Kit as previously described. Bio-Gide® collagen membrane (Giestlich Pharma, France) was used as a control for cell proliferation experiments.

#### 2.7.4 Cell Morphology and Tissue Colonisation

After 15 days of culture on d-WJ, WJ-SCs were fixed with 4% (w/v) paraformaldehyde (Sigma-Aldrich) at 37°C for 10 min. For morphology evaluation, cells were then permeabilized with 0.5% (v/v) Triton X-100 for 5 min. Alexa® Fluor-488 conjugated-Phalloidin® (1/100 dilution in 0.1% Triton X-100) was used to stain F-actin for 45 min at room temperature. Nuclei were counter-stained with 4,6-diamidino-2-phenylindole (DAPI, 100 ng/ml, 1/3 000 dilution) for 5 min. Stained cells were mounted and imaged by confocal laser scanning microscopy (CLSM, Zeiss LSM 710 NLO, 20× objective, Numerical Aperture 1.4, and Germany). For tissue colonisation evaluation, samples were embedded in paraffin and cut into 4 μm sections and HES staining was performed as previously described.

#### 2.7.5 Subcutaneous Implantation

All animal studies were carried out following the guidelines approved by the Committee on Animal Care Bourgogne Franche-Comté University (N° 2010-2206-02314). *In vivo* biological response to d-WJ was assessed with immunocompetent 8 weeks males Wistar rats (*n* = 4). UV-decontaminated (30 min) d-WJ membranes were cut with a scalpel into some pieces weighing around 10 mg. The d-WJ membranes were subcutaneously sutured and each rat received two d-WJ (according to 3R rules). After 3 weeks of implantation, the rats were sacrificed and the implanted membranes and the surrounding connective tissues and skin were resected from the underlying muscles. Samples were fixed in 4% paraformaldehyde, embedded in paraffin and cut into 4 μm sections as previously described. HES and MT staining were performed separately on consecutive tissue sections and images were taken using VS 120 OLYMPUS scanner.

### 2.8 Statistical Analyses

All statistical analyses were performed using GraphPad Prism software. The efficiency of decellularization was confirmed with at least six independent umbilical cords. Biochemical and swelling properties were determined from four independent umbilical cords. Bioactivity and mechanical characterization of samples were performed with five and six independent umbilical cords, respectively. For bacterial studies, at least three independent bacterial preculture were carried out for each bacterial strain and all samples were tested for each preculture in triplicate. All results were represented as histograms (mean ± SEM), statistical analysis were performed using Mann & Whitney test. For each test, a value of *p <* 0.05 was accepted as statistically significant *p* (rejection level of the null-hypothesis of equal means).

## 3 Result and Discussion

Healthy perinatal tissues are promising biological scaffolds as they are inexpensive, and universally available. Among these tissues, the human umbilical cord is expected to offer outstanding assets for tissue engineering by serving as a bioactive platform for supporting endogenous cell adhesion, growth, and differentiation. (Beiki et al., 2017; Jadalannagari et al., 2017; Kehtari et al., 2019; Penolazzi et al., 2020). Herein, the functional and technical justification for using decellularized WJ (d-WJ) as a biodegradable matrix instead of viable WJ tissue resides from its versatility and flexibility of use as an advanced biological scaffold with a wide range of applications, surpassing thereby the limitations of donor shortage and adverse host reaction. After stripping the surrounding superfluous tissues (*i.e.* sub-amniotic envelop and blood vessels) and technical optimization, the selected decellularization consisted of a combination of Triton X-100 (a milder non-ionic detergent, which targets the lipid–lipid and lipid–protein interactions, leaving the protein–protein interaction intact) and nuclease (DNase). For further clinical and commercial benefit (*i.e.* ease of transportation logistics and storage requirements), (Moore et al., 2017), d-WJ samples were freeze-dried. The physical, mechanical and biological properties of d-WJ were compared to those obtained for devitalized WJ control.

### 3.1 Structural Integrity

**FIGURE 1:**
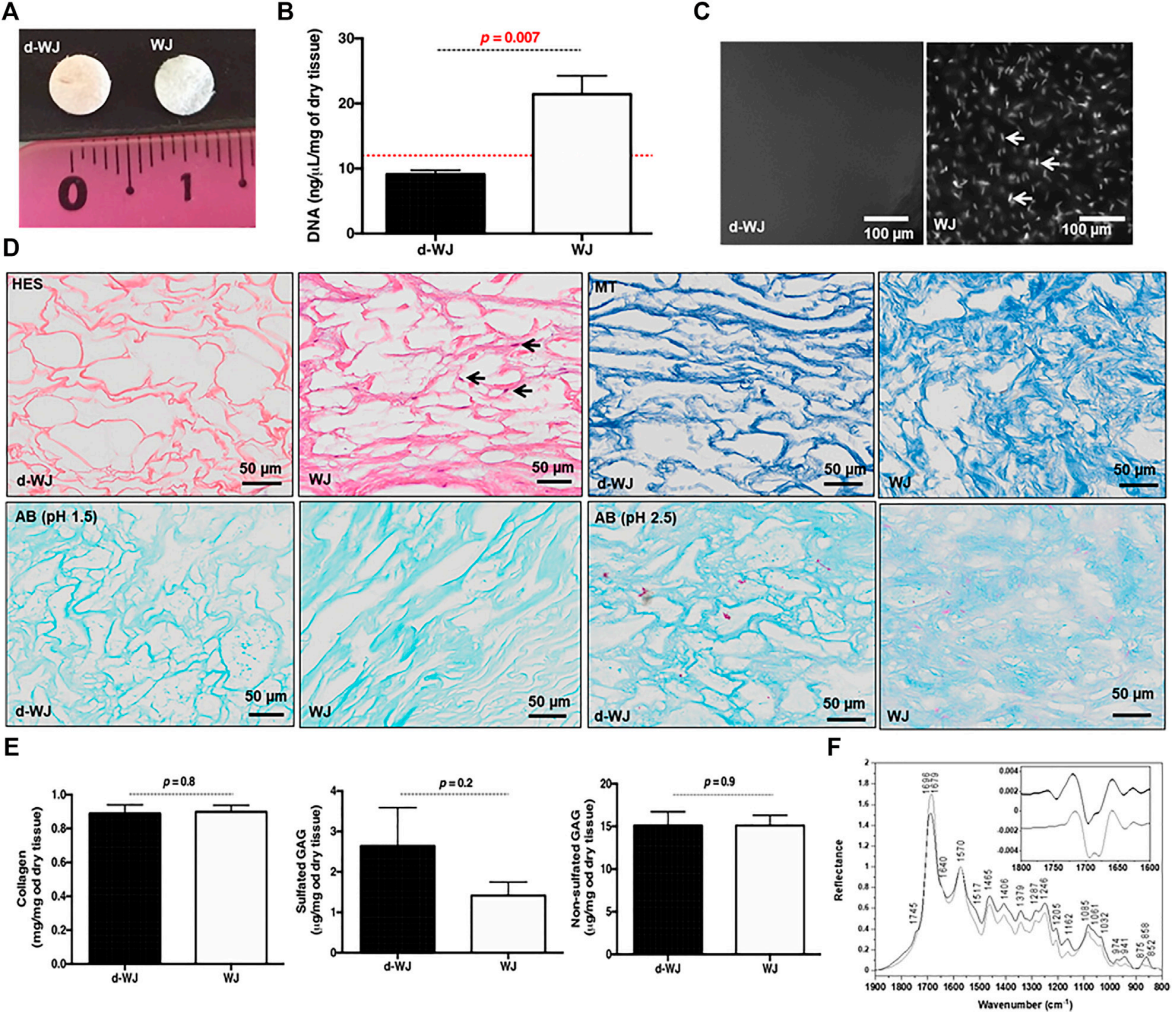
Structural characterization of d-WJ*.* **(A)**: Macroscopical view of d-WJ and WJ control. **(B)**: Nuclei moieties quantification in d-WJ and WJ, indicating a significant decrease in DNA content within d-WJ (red dashed line indicates the limit of detection of the used kit; *n* = 6, Mann & Whitney test). **(C)**: Fluorescence microscopy observation of DAPI stained nuclei samples, indicating the absence of nuclear structures in d-WJ and the presence of nuclei in WJ samples as highlighted by white arrows (scale bars = 100 μm). **(D)**: Hematoxylin-eosin-safran (HES, in upper left, black arrows highlighting stained nuclei), Masson’s trichrome (MT in upper right), Alcian Blue (AB at pH 1.5 in lower left and at pH 2.5 in lower right) of paraffin embedded d-WJ and WJ control (scale bars = 50 μm). **(E)**: Biochemical assays for collagen, sulphated and non-sulphated glycosaminoglycans, indicating a preservation in ECM components within d-WJ (*n* = 4, Mann & Whitney test). **(F)**: Infrared (IR) micro-spectroscopy spectra of d-WJ (grey) and WJ control (black) Spectra are normalized to one for the amide II band at 1,570 cm^−1^. Insert represents second derivative spectra of d-WJ (grey) and WJ control (black) in the 1800–1,600 cm^−1^ region.

The effective removal of cells and nucleic moieties from WJ (Figure 1A) was assessed by DNA quantification and histological staining (Figures 1B–D). The nucleic acids were hardly distinguished in d-WJ as indicated by hematoxylin-eosin-safran (HES) (Figure 1D) and 4,6-diamidino-2-phenylin-dole (DAPI) (Figure 1C) staining and corroborated by DNA content analysis (Figure 1B). Extracted DNA from d-WJ was 9.12 ± 1.41 ng/μl, below the limit detection of the used kit (*i.e.* 12 ng/μl), while extracted DNA from WJ was 21.42 ± 6.35 ng/μl/mg of dry tissue. Thus, the total DNA content of WJ was 664.12 ± 210.75 ng/mg of dry tissue which is above the threshold of 50 ng/mg (Crapo et al., 2011). These results indicate a successful removal of nuclei components from the d-WJ using Triton X-100 and DNase treatments.

Preservation of the ECM integrity after decellularization was a paramount goal in this study. Herein, the ECM components (*i.e.* total collagen and sulphated and non-sulphated glycosaminoglycans (GAGs)) were analysed qualitatively and quantitatively. Qualitative Masson’s Trichrome and Alcian Blue staining showed that d-WJ resulted in primarily collagen and both sulphated and non-sulphated GAGs (Figure 1D). The quantitative biochemical investigations indicated that d-WJ composition is consistent with the ones reported for WJ as a great amount of collagen (0.88 ± 0.09 *vs* 0.89 ± 0.06 mg/mg of dry tissue), sulphated GAGs (3.14 ± 1.50 *vs* 1.6 ± 0.62 μg/mg of dry tissue) as well as non-sulphated GAGs (15.09 ± 3.29 *vs* 15.11 ± 1.94 μg/mg of dry tissue) was noticed (Figure 1E). To sum up, our results suggest that the used decellularization method fully meets modern criteria of decellularization. (Crapo et al., 2011; Keane et al., 2015).

Chemical composition of d-WJ was measured by infrared (IR) micro-spectroscopy (Figure 1F). The band assignments, gathered in Table 1, were performed with the help of the second derivative spectra, and in accordance with the literature. (Cortizas and López-Costas, 2020; Belbachir et al., 2009; Petibois et al., 2006; Foot and Mulholland, 2005; Gough et al., 2003; Haxaire et al., 2003; Chirgadze et al., 1975). In comparison with WJ, d-WJ spectra revealed the absence of phospholipid shoulder (located at 1745 cm^−1^, corresponding to cell components) along with a reduction in bands intensities close to 1,240 and 1,085 cm^−1^ (partially assigned to PO_2_ stretching bands from phospholipids and nucleic acids), confirming the effectiveness of the decellularization. The effect of the tissue processing on the ECM integrity was also investigated. The general shape of the spectra was close to those already reported in the literature for collagen. (Cortizas and López-Costas, 2020; Petibois et al., 2006). Indeed, both d-WJ and WJ control spectra showed major bands around 1,685 and 1,570 cm^−1^, assigned mainly to amide I and II bands from collagen, respectively. Amide I band is very sensitive to the secondary structure of collagen. Two main bands were resolved at 1,696 and 1,679 cm^−1^ in the second derivative spectra (Figure 1F insert), assigned to β-turns and β-sheets of collagen, respectively, (Belbachir et al., 2009), revealing an apparent difference in their relative intensities following the decellularization. Indeed, ratio β-turns to β-sheets decreased from the WJ spectrum to d-WJ spectrum, suggesting a potential alteration of the collagen upon decellularization. Spectra also exhibit absorption bands between 1,170 and 800 cm^−1^, which arise mainly from the ν(C–O) and ν(C–O–C) absorptions of the carbohydrate moieties from collagen and GAGs. (Haxaire et al., 2003; Petibois et al., 2006). Spectra of d-WJ showed lower absorption intensities compared to WJ spectra, indicating a partial loss of GAGs content. Interestingly, GAGs were still left after decellularization as it is shown for example by the occurrence of the band at 852 cm^−1^, the latter being specific for chondroitin-4-sulphate. (Allen et al., 1977; Foot and Mulholland, 2005).

**TABLE 1:** Assignments of principal micro-infrared vibrational bands of the 1800–800 cm^−1^ region of the spectra of d-WJ and WJ.

Infrared wavenumber from 2nd derivative spectra (cm^−1^)		Tentative assignment	Main associated compound
d-WJ	WJ		
—	1745	νC = O (esters)	Phospholipids
1,696	1,696	Amide I	Collagen, β-turn
1,679	1,679	Amide I	Collagen, β-sheet
1,639	1,640	Amide I, δH_2_O	Collagen, α-helix and random, Water
1,614	1,614		β-sheets, amino acid side chains (Glu, Arg)
1,570	1,572	Amide II	Collagen
1,517	1,515	Amide II	β-sheets
1,465	1,465	δCH_n_	Collagen
1,406	1,406	νCOO^-^	Collagen, GAGs
1,380	1,379	δCH_3_	Collagen
1,343	1,343	δCH_3_, Amide III	Collagen
1,316	1,316	Amide III	Collagen
1,287	1,287	Amide III	Collagen
1,246	1,246	Amide III, νsSO_2_, and νsPO_2_	Collagen, GAGs, phospholipids and nucleic acids
1,205	1,205	Amide III	Collagen
1,162	1,162	νC-O	Collagen (carbohydrate moiety), GAGs
1,085	1,085	νC-O, νsPO_2_	Collagen (carbohydrate moiety), GAGs
1,061	1,061	νC-O	Collagen (carbohydrate moiety), GAGs
1,032	1,032	νC-O	Collagen (carbohydrate moiety, hydroxyproline), GAGs
974	975	C-O	Collagen (carbohydrate moiety)
941	941	C-O-C skeletal	Collagen (carbohydrate moiety)
875	875	C-C	Collagen
—	858	C-O-S	GAGs
852	852	C-O-S	GAGs (chondroitin-4-sulphate)

(Key: ν, stretching; δ, bending; ω, wagging; a, antisymmetric; s, symmetric; sh, shoulder; GAGs, glycosaminoglycans).

### 3.2 Physical and Mechanical Features

**FIGURE 2:**
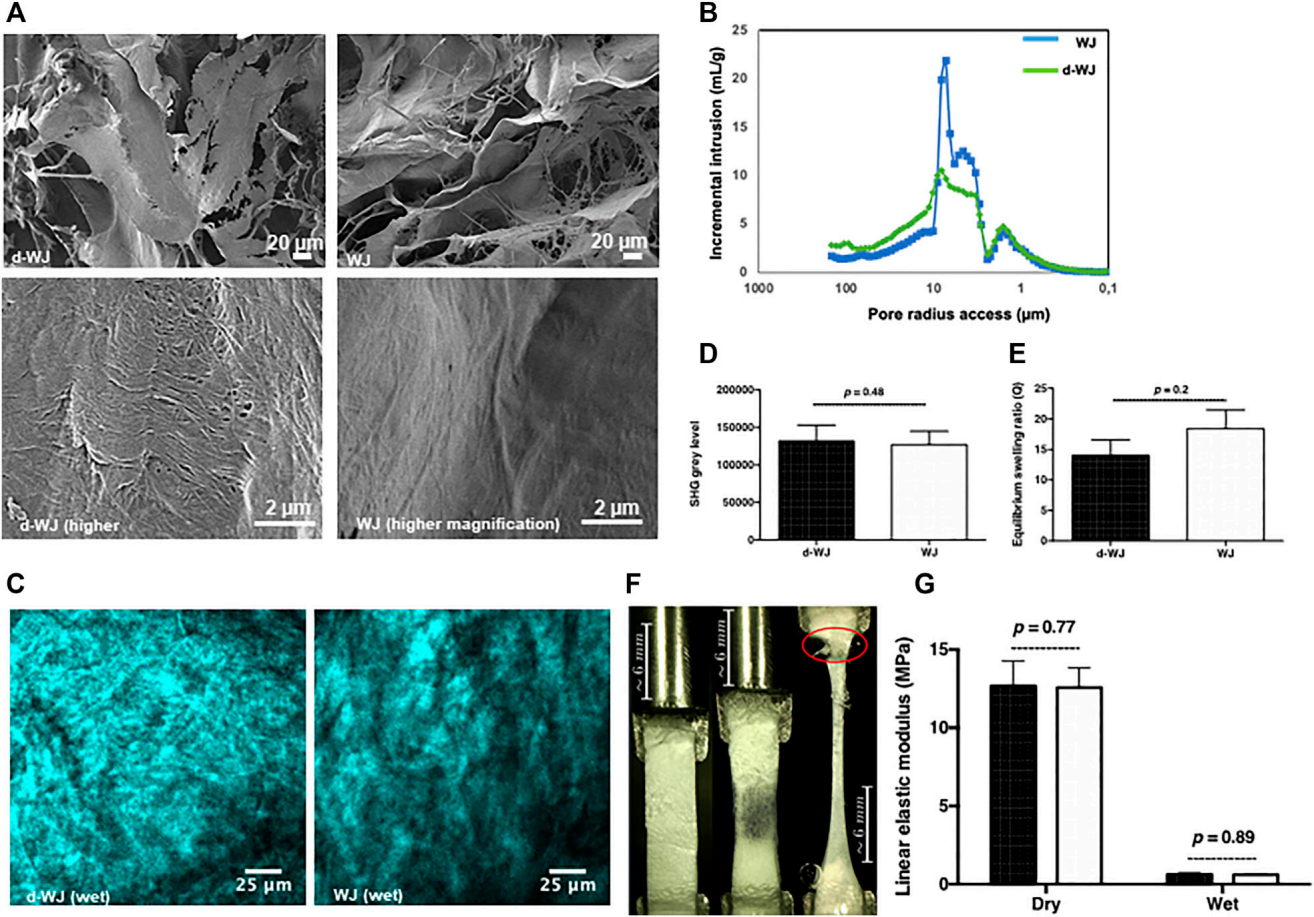
Physical and mechanical characterization of d-WJ. **(A)**: Scanning electron microscopy views at low magnification (in upper line) and high magnification (in lower line; scale bars = 20 and 100 μm, respectively). **(B)**: Mercure intrusion porosity, **(C,D)**: Two-photon excitation laser scanning confocal microscopy and second harmonic generation imaging (Scale bars = 25 μm) and signal quantification, respectively, indicating no alteration in collagen integrity within d-WJ (*n* = 4, Mann & Whitney test). **(E)**: Equilibrium swelling ratio of d-WJ in PBS, showing a preservation in hydration capabilities of d-WJ (*n* = 4, Mann & Whitney test). **(F)**: Mechanical testing photographs (in red is indicated the rupture at the fixation site) and **(G)**: Linear elastic modulus of d-WJ (black bars) in dry and wet experimental conditions, showing no noticeable difference in d-WJ mechanical response *versus* WJ (white bars; *n* = 6, Mann & Whitney test).

The firmness of the umbilical cord is related to the fibrous collagenic nature of WJ. (Converse et al., 2018). Herein, we investigated the collagen structure by scanning electron microscopy (SEM) and Two-photon excitation laser scanning confocal microscopy and second harmonic generation (SHG). SEM micrographs of the cross-section and surface of the d-WJ demonstrated the nanofibrous architecture of d-WJ with variable porosity, close to WJ features (Figure 2A). Deep amino acid composition of WJ, performed by ion exchange chromatography (data not shown), showed that WJ did not exhibit similar composition with fibrillar type I collagen. Thus, the collagen fibril periodicity of ∼100 nm could be attributed to type VI collagen while unspecific bending periodicity fibrils could be attributed to type V collagen. (Nanaev et al., 1997; Franc et al., 1998).

Mercury intrusion porosimetry method confirmed the highly porous microstructure of d-WJ with an average total porosity of about 78.9% close to the WJ total porosity (79.8%). However, the pore access distribution differed between the two samples. d-WJ showed a decrease in the main pore access between 3 and 9 μm while the amount of the larger pore access (>10 μm) increased (Figure 2B). Collagen orientation plays a crucial role in the structural and mechanical features of the tissue. SHG have seen a surge in use in biomedical research, allowing the simultaneous visualization of endogenous auto-fluorescent unstained samples and hyper-polarizable fibrillar protein. (Cicchi et al., 2013). Thus, 3D images of the organization of collagen fibers with micrometer resolution were given by polarized-SHG visualization. Despite the effect of decellularization on the secondary structure of collagen, d-WJ showed a strong SHG signal with a disordered structure close to WJ (Figures 2C,D). However, collagen fibres were hardly distinguished on our experimental setup (*i.e.* PBS hydrated tissues). Thus, the provided signal could be attributed to the centrosymmetric type I, type III and type VI collagen. (Ricard-Blum et al., 2018; Reed et al., 2019). The diminished SHG in decellularized porcine urinary bladder being correlated to a strong disruption of the collagen structure, (Hwang et al., 2017), SHG signals from d-WJ are thus indicative of the absence of structural damages in the fibrillar collagen.

Water absorption capacity is one major consideration in biomedical field. WJ is a porous matrix with canalicular structures occupied mainly by hyaluronic acid. (Sobolewski et al., 1997). Therefore, the interaction with water forms a highly viscous fluid within the pore space of the matrix. (Ferguson and Dodson, 2009). To address how much and how quickly matrices absorb the fluids from their surroundings, swelling properties of the d-WJ were evaluated. Water absorption reached the equilibrium swelling within 5 min. By calculating the swelling ratio, a slight decrease was observed for d-WJ in comparison with WJ (14.0 ± 6.7 and 18.5 ± 9.2, respectively; *p =* 0.2; Figure 2E), confirming the preservation of GAGs content following the decellularization.

From its structural basis point of view (*i.e.* collagen, sulphated and non-sulphated GAGs), the highly hydrated WJ possesses a strong mechanical resistance and elasticity. (Pennati, 2001). The linear elastic modulus, a linear approximation of the stress strain curve translating the linear elastic response of the tissue, was monitored in dry and wet conditions for an average strain load of 1.59% to avoid sample damage. The results showed a decrease in linear elastic modulus from 12.69 ± 3.87 MPa in dry condition to 0.64 ± 0.22 MPa in wet condition for d-WJ and a decrease from 12.57 ± 3.08 to 0.61 ± 0.11 MPa in wet condition for WJ (Figures 2F,G). Previous studies reported an increase in tissue stiffness following the decellularization, because of the relative increase in collagen content and reduction of other components (*i.e.* water and GAG). (Totonelli et al., 2012; Frazão et al., 2020). The linear elastic modulus of d-WJ close to WJ is in accordance with the above cited biochemical characterization. Altogether, these results demonstrated that by eliminating the cell and nuclei moieties, the macrostructure and the mechanical integrity of d-WJ was not compromised.

### 3.3 Degradation

Biodegradability is an important factor for matrices designing in regenerative medicine. Collagenase and hyaluronidase are usually used to partially mimic the *in vivo* biodegradation conditions for collagen and hyaluronic acid based tissues such as cartilage. (Lau et al., 2019). Regarding the composition of WJ, herein, the effect of decellularization on WJ degradation was examined by treating d-WJ with collagenase, hyaluronidase, and enzyme free-phosphate buffer solution (PBS). As for WJ, d-WJ was completely degraded in collagenase after 72 h of incubation since a negligible amount was harvested. At 48 h of incubation, samples retained 8 ± 2% of collagen for d-WJ and 14 ± 4% for WJ (*p =* 0.2). Samples maintained in hyaluronidase and PBS did not show a loss in their weight over a week (Table 2) *in vivo* (Lau et al., 2019). Herein, collagenase and hyaluronic acid were used in much higher concentrations than found in physiological conditions, but they allowed to accelerate the biodegradation and to observe the effect in shorter time. Our results revealed that d-WJ was sensitive to the collagenase action but seemed resistant to hydrolysis probably due the presence of insoluble collagen constituents. (Sobolewski et al., 1997). The absence of weight loss in hyaluronidase could be attributed to the low content in hyaluronic acid in WJ matrix (several μg) in comparison with collagen (several mg) (Figure 1E). To verify the resistance of WJ degradation in hyaluronidase, we quantified the remanent non-sulphated GAGs in samples after 2 h of incubation. Our results showed that only 29 ± 13% of non-sulphated GAGs was retained in d-WJ *versus* 49 ± 13% in WJ (*p =* 0.2). Despite the increase in pore size in d-WJ that could induce a higher diffusion of enzymes within the tissue, our results did not reveal a major effect of the decellularization on the *in vitro* matrix biodegradability.

**TABLE 2:** *In vitro* degradation testing in collagenase, hyaluronidase, and in phosphate buffer.

Conditions	Time of incubation (h)	d-WJ (%)	WJ (%)
Weight lost following incubation in PBS	72	neglecting	neglecting
Remaining weight following collagenase treatment	48	8 ± 2^NS^	14 ± 4
Weight lost following hyaluronidase treatment	72	neglecting	neglecting
Remaining non-sulphated GAGs following hyaluronidase treatment	2	29 ± 13^NS^	49 ± 13

(GAGs, glycosaminoglycans; PBS, Phosphate buffer solution; NS, non-significant difference, *n* = 6, Mann & Whitney).

### 3.4 Bioactivity

**FIGURE 3:**
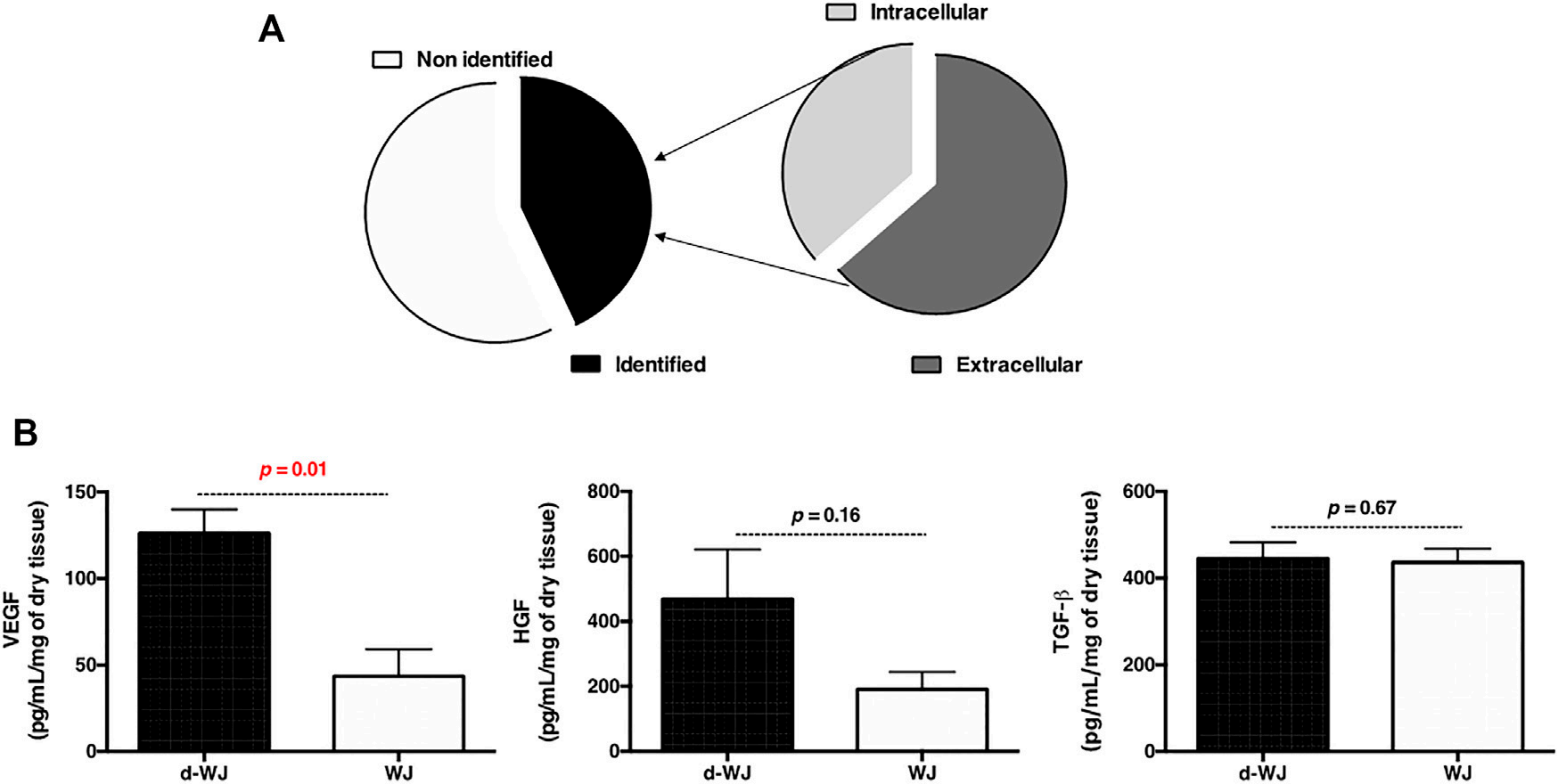
Bioactivity of d-WJ matrix*.* **(A)**: Mass spectrometry analysis of released macromolecules in d-WJ supernatant. **(B)**: ELISA analysis of released growth factors, showing an increase growth factors release from d-WJ (*n* = 5, Mann & Whitney test).

WJ holds great potential in treating a variety of chronic wounds with a minimal scar formation. (Hanselman et al., 2015). Few empirical clinical studies have reported that transplantation of umbilical cord facilitates epithelial wound healing and reduces inflammation, scarring, and angiogenesis. (Marston et al., 2019; Tettelbach et al., 2019; Caputo et al., 2016). Macromolecules derived from WJ-ECM are believed to promote resolution of inflammatory processes, to support stromal cell recruitment, and to inhibit myofibroblasts. (Gupta et al., 2020). Herein, we supposed that when WJ is exposed to the biological fluid, most of the permeable molecules immediately enter the fluid due to the osmotic pressure, increasing the tissue bioactivity. In this context, to detect the bioactivity of d-WJ, tissues were soaked in bovine serum free α-MEM and bovine serum supplemented α-MEM culture media for 72 h and the released macromolecules and growth factors were respectively analysed by mass spectrometry and ELISA. Mass spectrometry showed a passive release of structural and adhesive proteins involved in wound healing process and antimicrobial response such as collagen, fibronectin, tenascin, lumican, periostin, Keratin type I and II, Fibulin and Fibrinogen beta chain (Figure 3A). Interestingly no spontaneous release of these proteins was detected for WJ. The obtained mass spectrometry results are in accordance with Jadalannagari *et al.*, who detected these molecules after WJ matrix degradation. (Jadalannagari et al., 2017). The retention of growth factors naturally occurring in the d-WJ was demonstrated by ELISA analysis of culture medium. Vascular endothelial growth factors (VEGF), hepatic growth factors (HGF) and transforming growth factors beta (TGF-β) were detected in the supernatant (Figure 3B). An increase in VEGF and HGF release in the culture medium was noticed for d-WJ in comparison with WJ. Our results suggest that d-WJ is more exposed to the culture medium diffusion into WJ matrix, allowing the release of bioactive molecules. The detection of the released molecules in d-WJ could be due to the increase in the pore size following the decellularization. Furthermore, it is well known that growth factors bind to sulphated GAG compound(s) such as heparan sulphate, chondroitin sulphate to form complexes with ECM components. In our experiments, the growth factors were not detected in the absence of bovine serum. Therefore, we concluded that the action of proteases and/or glycosidases naturally present in bovine serum may release and activate growth factors within the tissue. Taken together, these results suggest that d-WJ are bioactive matrix, suitable for regenerative medicine strategies.

### 3.5 Antibacterial Activity

**FIGURE 4:**
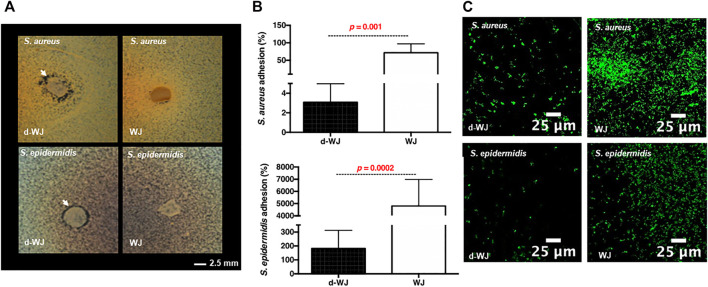
Antibacterial properties of d-WJ. **(A)**: Agar diffusion test, white arrows indicate zone of bacterial growth inhibition of *S. aureus* and *S. epidermidis* around d-WJ. **(B)**: percentage of adhered bacteria on d-WJ matrix, and **(C)**: Confocal laser microscopy visualization of Syto-9® labelled bacteria on d-WJ matrix (green color), indicating a significant decrease in bacteria adhesion on d-WJ (*n* = 9, Mann & Whitney test, scale bars = 25 μm).

As mentioned previously, WJ shows promising results in clinic following treatment of diabetic ulcers with osteomyelitis. (Marston et al., 2019; Tettelbach et al., 2019; Caputo et al., 2016; Raphael, 2016). Osteomyelitis is most commonly caused by the *Staphylococcus aureus* (*S. aureus*) and *epidermidis* (*S. epidermidis*), opportunistic pathogens responsible for a tremendous burden on the healthcare system. One of the reasons that Staphylococci are problematic is their well-known ability to attach to surfaces and develop into recalcitrant community structures, often referred to as a “biofilm”. Mass spectrometry revealed the passive release of antimicrobial molecules involved in the bacterial agglutination (*i.e.* Fibrinogen beta chain). (Hanington and Zhang, 2011; Sun et al., 2014). Consequently, we evaluated the antibacterial effect of d-WJ against *S. aureus* and *S. epidermidis* strains. Performed agar diffusion test highlighted the presence of a zone of microbial growth inhibition for both pathogens in contact with d-WJ, while WJ did not demonstrate any inhibition zone (Figure 4A). These results suggest a rapid diffusion of antimicrobial molecules from d-WJ such as fibrinogen beta chain Bacterial adhesion to d-WJ was then evaluated and showed a clear inherent antiadhesive effect of d-WJ in comparison with WJ (Figures 4B,C). On d-WJ, adherent *S. aureus* and *S. epidermidis* were respectively 23 and 26 times less than that on WJ (*p <* 0.001). Taken together, these results suggest that d-WJ had an intrinsic antibacterial effect against *Staphylococcus* strains. In addition to the release of antibacterial agents from d-WJ, we thought that the hyaluronic acid (Marcuzzo et al., 2017) component of the d-WJ extracellular matrix might be responsible for the reduction of the bacterial adhesion. Further experiments are needed to decipher the exact antibacterial action of d-WJ against *Staphylococcus* strains.

### 3.6 Biocompatibility *in vitro* and *in vivo*

**FIGURE 5:**
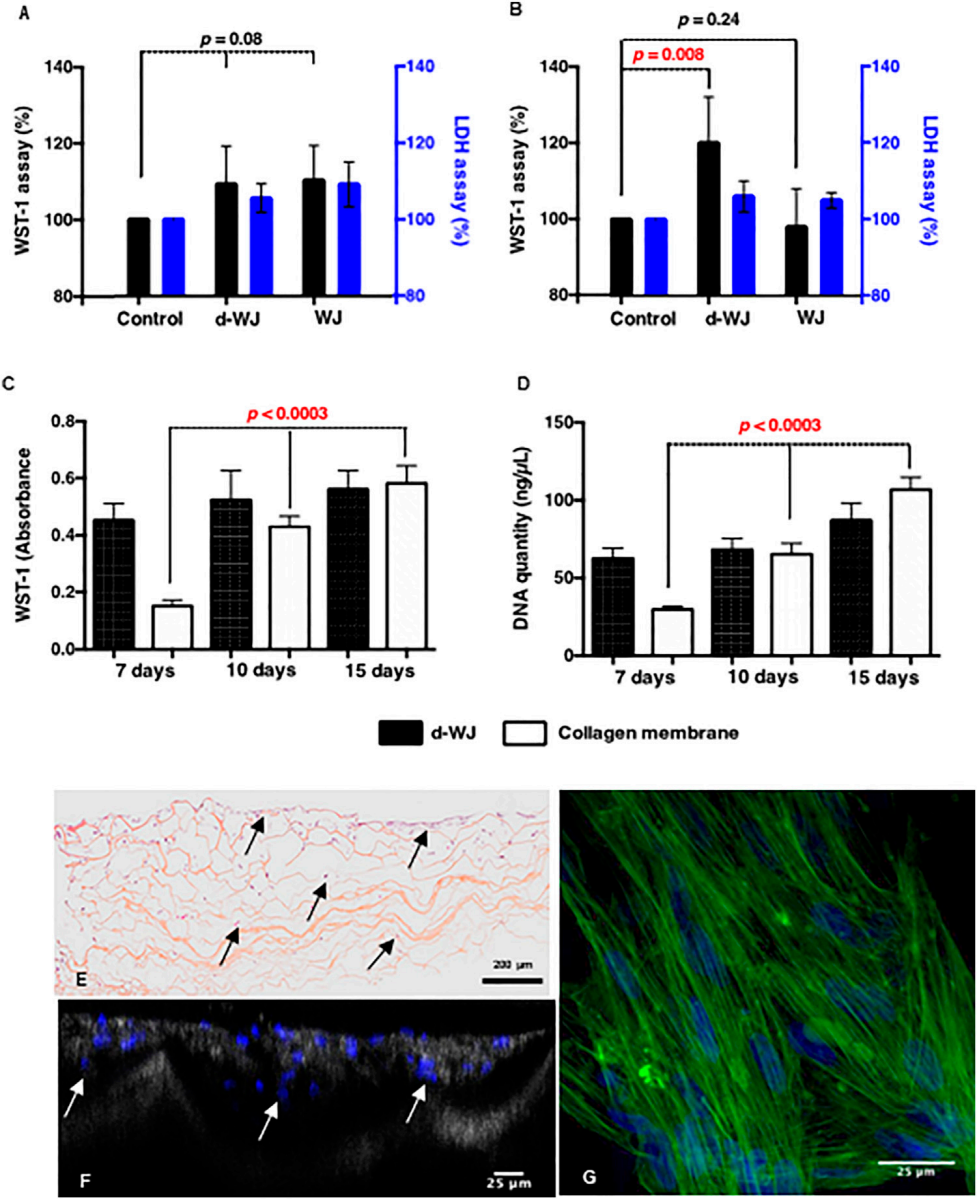
In vitro biocompatibility of d-WJ. **(A,B)**: Metabolic activities (black bars) and LDH release (blue bars) of WJ-SCs and fibroblasts, respectively in the presence of d-WJ and WJ control, indicating the absence of cytotoxic release of Triton X-100 from d-WJ (*n* = 6, Mann & Whitney test). **(C)**: WJ-SCs proliferation assay and **(D)**: DNA quantification of WJ-SCs cultured on d-WJ (black bars) and Bio-Gide® positive control (white bars), showing the absence of WJ-SCs proliferation on d-WJ (*n* = 6, Mann & Whitney test). **(E, F)**: Cross sections of WJ-SCs cultured on d-WJ stained respectively with HES and DAPI. Arrows indicate cells within the d-WJ. **(G)**: Confocal microscopy view of WJ-SCs cultured on d-WJ labelled with phalloidin (in green) and DAPI (in blue). Scale bars = 200 μm **(E)** and 25 μm **(F,G)**.

Decellularized allografts are classified by regulatory agencies FDA as a human cell or tissue product and therefore does not require investigational new drug or device exemption approval. (Moore et al., 2017; Sutherland et al., 2015). However, they require biocompatibility testing prior to the clinical use. (Hussein et al., 2016). Consequently, the cytocompatibility d-WJ was investigated. WJ derived stromal cells (WJ-SCs) and gingival fibroblasts were cultured in 24 well chamber culture for 24 h, then 1 mg of either d-WJ or WJ was added in culture well. WJ-SCs and fibroblasts cultured on tissue culture plastic in the absence of WJ samples were used as controls. According to the ISO/EN 10993 part 5 guideline, the cytotoxicity of d-WJ was monitored by WST-1® (water-soluble tetrazolium salt-1®) assay and LDH (lactate dehydrogenase) release in culture supernatants. When compared to plastic positive control, both WJ-SCs and fibroblasts cultured in the presence of d-WJ remained above 70% of cell viability, threshold considered as an indicator of cytotoxic phenomenon. Furthermore, no increase in the LDH release was observed (Figures 5A,B). Taken together, our results indicated the absence of toxic agents release (*i.e.* Triton X-100) following the tissue processing. It is generally accepted that an increase in the WST-1® absorbance at 450 nm reflects a proliferating state of cells. Herein, we noticed an increase in fibroblast metabolic activity in the presence of d-WJ, while no increase in the release of LDH was observed, and suggesting a potential increase in fibroblast proliferation (Figure 5B). This proliferation could be attributed to the released growth factors from d-WJ, confirming thus its bioactive features. Preparing an appropriate matrix with good biocompatibility and mechanical properties that mimics the native microenvironment of cells is one of the key elements in regenerative medicine. It is known that there is a broad range of interactions between mesenchymal stromal cells and their niche, ensuring the stromal cell’s function. (Shakouri-Motlagh et al., 2017). WJ-SCs possess regenerative capability close to the bone marrow derived stromal cells. The question remains unanswered whether the d-WJ could be recolonized by WJ-SCs. Therefore, WJ-SCs were cultured on d-WJ at a density of 10^4^ cells/5 mm diameter of samples. Clinical device collagen membrane (Bio-Gide®) was used as a control. The cell proliferation was monitored by WST-1® assay and DNA quantification at day 7, 10, and 15 of culture, using independent samples for each test and time point. While WST-1® and DNA quantification did not show significant variation of each test and time point in d-WJ (*p =* 0.7), while an increase in cell content was observed on the Bio-Gide® positive control (*p <* 0.0003) over the studied time (Figures 5C,D).

Despite the small pore size of d-WJ (ranging from 10 to 184 μm), histological section and laser scanning confocal microscopy showed the presence of WJ-SCs within d-WJ (Figures 5E,F). WJ-SCs morphological examination of adhered WJ-SCs on d-WJ showed elongated cells on the top of d-WJ (Figure 5G). To sum up, these results suggest that d-WJ could simulate the natural niche and microenvironment of WJ-SCs, bringing them closer to their best function similar to what they do in the body. Thus, d-WJ could serve as a platform for WJ-SCs delivery.

**FIGURE 6:**
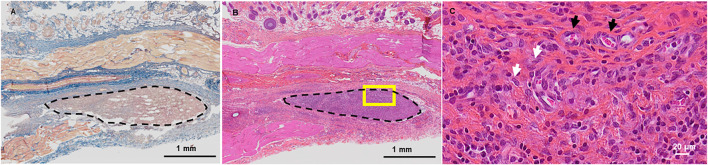
In vivo Biocompatibility of d-WJ. **(A)**: Masson’s Trichrome staining. **(B, C)**: Hematoxylin-eosin-safran staining. Dashed delimitations indicate the implanted d-WJ. Picture in C represents a higher magnification of the yellow square in **(B)**. White and black arrows indicate fibroblasts and blood vessels, respectively. Scale bars = 1 mm **(A,B)** and 20 μm **(C)**.

Finally, we sought to investigate *in vivo* the tissue response of the recipient organism following d-WJ implantation. Thus, d-WJ was tested according to ISO/EN 10993 part 6 guidelines, by using subcutaneous implantation. Perinatal tissues including the umbilical cords are described as “unrecognized as a foreign material”. (Hanselman et al., 2015; Ramuta et al., 2021). We have thus intentionally chosen immunocompetent rat for this study, in order to be able to take immunological reactions into consideration. In all animals, wounds were closed with no wound infection or other complications. Hair growth was normal in the operation field. No inflammatory response, no connective tissue capsule and good histocompatibility were noticed at the studied time point; d-WJ was completely replaced with the reparative granulation tissue. In addition, multinucleated giant cells were not found around the implanted d-WJ. Masson’s Trichrome staining did not indicate the presence of collagen matrix, suggesting the total degradation of the implanted d-WJ (Figure 6A). The healing process involves many closely coordinated regenerative reactions, counting hemostasis, the migration of fibroblasts and endothelial cells, inducing the tissue remodeling and degradation of collagen fibers. (Stoica et al., 2020). HES staining revealed the presence of blood vessels and fibroblast-*like* cells (Figures 6B,C). These results confirmed the *in vitro* and *in vivo* biocompatibility of d-WJ.

## 4 Conclusion

The present work showed that a fully decellularized matrix can be obtained from human Wharton’s jelly. Decellularized WJ matrix exhibited overall desirable tissue engineering characteristics. Decellularization process increased the bioactivity of the WJ matrix, inducing a significant release of angiogenic VEGF as well as a passive release of macromolecules with an antibacterial effect against *S. aureus* and *S. epidermidis* strains. Further researches are needed to elucidate which WJ derived macromolecules cause the potent antibacterial effect. Biodegradability and stromal cell homing capabilities reinforced with excellent mechanical and physical properties render d-WJ ideal for stem cell therapy and stem cell delivery applications. In large scale future studies, the investigation of the potential use of d-WJ as an antibacterial therapeutic dressing for scar-free wound healing might promote faster and safer translation of d-WJ into clinical practice. Furthermore, the hybridization of autologous blood product such as platelet rich plasma (Barkestani et al., 2021) or autologous stem cells could increase the bioactive potential of d-WJ (Aubert et al., 2017).

## Data Availability

The raw data supporting the conclusion of this article will be made available by the authors, without undue reservation.
